# Projected Cost Savings of a Community Health Worker Model for Asthma Home Visits in the Massachusetts Pediatric Medicaid Population

**DOI:** 10.5888/pcd21.240028

**Published:** 2024-09-12

**Authors:** Maya Mahin, Jing Guo, Michelle Warner, Maya Dottin, Nina Olsen, Erica T. Marshall

**Affiliations:** 1 Massachusetts Department of Public Health, Boston

## Abstract

**Introduction:**

The community health worker–led asthma home visiting model (CHW model) improved asthma outcomes and reduced health care costs among Massachusetts children with asthma. We projected cost savings associated with the expansion of the CHW model among pediatric Massachusetts Medicaid (MassHealth)-eligible patients with uncontrolled asthma (≥2 asthma-related emergency department visits per year).

**Methods:**

We estimated 2019 costs associated with asthma-related hospitalizations and emergency department visits for MassHealth pediatric patients with uncontrolled asthma who also had 365 days of Medicaid eligibility in 2019. We based estimated cost savings on previously published results from a study of a comparable patient population.

**Results:**

The projected asthma-related cost savings from expansion of the CHW model were $566.58 per patient, or $774,514.86 total, for the 1,367 MassHealth-eligible children with uncontrolled asthma in our analysis.

**Conclusion:**

Expansion of the CHW model is an effective way to increase asthma services and reduce Medicaid costs for MassHealth patients, a population made up disproportionately of Black and Hispanic residents with low incomes.

**Summary**

**What is already known on this topic?**

Interventions for children with asthma that include home visits by community health workers (CHWs) can improve asthma-related health and economic outcomes.

**What is added by this report?**

By using 2019 claims data, we projected annual savings per patient in health care costs associated with expansion of the CHW-led asthma home visiting model to all pediatric Massachusetts Medicaid (MassHealth)-eligible patients with uncontrolled asthma.

**What are the implications for public health practice?**

Expanding the CHW model to all MassHealth-eligible pediatric patients with uncontrolled asthma can improve asthma outcomes and save costs. By increasing asthma services for Black and Hispanic residents with low incomes, expansion may also reduce disparities in asthma outcomes.

## Introduction

Massachusetts has a high pediatric asthma burden. In 2021, 9.7% of all Massachusetts children had current asthma compared with 6.5% of US children overall (1). For the combined period from 2019 to 2021, asthma was categorized as “not well controlled” or “very poorly controlled” (2) in almost 40% of children with current asthma, based on self-reported data on factors associated with asthma management from the Massachusetts Behavioral Risk Factor Surveillance System Child Asthma Call-back Survey (3). Inequities exist in the use of asthma-related health care among the Massachusetts population. By using combined data from 2019 through 2021, the rates of asthma-related emergency department (ED) visits for Hispanic and non-Hispanic Black children aged 19 years or younger were significantly higher than the rates for non-Hispanic White children (4). Additionally, in 2018, the age-adjusted rates of asthma-related hospitalizations and ED visits for the combined population of children and adults in Massachusetts were approximately 3 to 4 times higher among non-Hispanic Black and Hispanic residents than non-Hispanic White residents (5,6). Similar inequities in pediatric asthma outcomes also exist nationally (7): by using combined data from 2018 through 2020, among children with current asthma, the percentage of children whose asthma was uncontrolled was approximately 7 to 10 percentage points higher among Black and Hispanic children than White children (7).

Asthma self-management — the ability of individuals and families to effectively manage asthma symptoms — is a vital component of asthma control. Structural racism and other systems of oppression contribute to inequities in health-related social needs (eg, food, housing, income, transportation), which too often lead to inequitable access to adequate health care and barriers to asthma control (8). Black and Hispanic children and their caregivers are often more likely than their White counterparts to have gaps in knowledge, skills, resources, and support for asthma self-management (9,10). Community health workers (CHWs) can help fill these gaps by providing culturally aware asthma self-management education and resources as a component of asthma home-visiting programs (11). Because of their lived experience in the communities they serve, CHWs are uniquely able to provide linguistically and culturally appropriate care. They play an important role by helping to address health-related social needs and by understanding the social determinants of health that affect their communities.

The health and economic benefits of the CHW model, a multicomponent, low-cost, asthma home visiting intervention for children with asthma and their caregivers, have been well established (11,12). These include an increase in the number of symptom-free days, a decrease in asthma-related health care use (eg, hospitalizations, ED visits), reduced exposure to environmental triggers, and improved caregiver quality of life (12–15). The CHW model is an important component of a comprehensive approach to reducing asthma-related racial disparities — racial inequities in asthma outcomes and inequities in conditions that cause asthma and make it more difficult to manage. The model can help reduce some of the barriers that families, particularly Black and Hispanic families, face in accessing culturally relevant and linguistically appropriate asthma care, barriers that result from the continuing structural racism that operates in the health care system (16,17).

In addition to assessment and education about the impact of the home environment on a child’s asthma, CHWs provide asthma services in the home. Doing so removes transportation-related barriers, making it easier for Black and Hispanic families that disproportionately live far from health care centers or must navigate public transportation systems to receive asthma services (18,19). The CHW model also increases the time spent receiving asthma care.

Despite a well-established evidence base, the number of CHW-led asthma home-visiting programs available in Massachusetts is limited, in part because sustainable financing for such services is not well established (20). However, a potential funding mechanism may be emerging: the recent 2023 MassHealth 1115 Demonstration waiver includes a potential sustainable funding mechanism for CHWs who support innovative service delivery models (21).

The objective of our study was to identify the hypothetical financial impact of a large-scale expansion of the CHW model across the MassHealth-eligible population of children with uncontrolled asthma, defined as 2 or more asthma-related ED visits per year, by quantifying the associated reduction in asthma-related health care costs. Based on the available evidence that the CHW model is associated with better asthma control (11), we also expected that these estimated cost reductions would be associated with improvements in asthma outcomes. Although published studies (11,12) have looked at the financial impact of the CHW model in small groups of patients with asthma, our study estimated the potential cost savings resulting from a hypothetical large-scale expansion of the model throughout the pediatric Massachusetts Medicaid eligible population. Our goal was to provide evidence to support efforts to establish sustainable financing mechanisms for expansion by demonstrating the potential cost savings associated with the CHW model.

## Methods

### Data source

Our primary data source was eligibility and medical insurance claims data for 2019 from MassHealth’s internal data warehouse, a clearinghouse of MassHealth medical claims and eligibility data that uses the IBM Cognos Analytics (IBM Corp) software platform for data access and querying. Data elements, including patient demographics, MassHealth eligibility, and information on specific services provided (eg, diagnosis codes, place of service, date of service, amount paid by MassHealth) are stored in discrete, structured fields (race and ethnicity data fields were largely incomplete in this data set.) The Massachusetts Executive Office of Health and Human Services approved and coordinated access to the data warehouse for our project team. The team extracted relevant data elements from the data warehouse by using the IBM Cognos reporting tool and transferred the data to SAS Studio (SAS) for analysis.

### Study population

The study population we identified for our analysis consisted of MassHealth-eligible pediatric patients with uncontrolled asthma (N = 1,367), defined as children aged 17 years or younger who had at least 2 asthma-related ED visits in 2019 where asthma was the primary or secondary diagnosis. To ensure that a complete set of calendar-year claims was included in our analysis, we further restricted our study population to children who had a full 365 days of MassHealth eligibility in 2019. We considered only ED visits with a primary or secondary diagnosis of asthma to ensure a high level of confidence that the visits were related to the child’s asthma control status. The Massachusetts Department of Public Health (MDPH) institutional review board determined that our study did not constitute human subject research according to federal regulations and did not require further review.

In our analysis, we calculated the potential cost savings of expanding the CHW model to our study population of 1,367 children with uncontrolled asthma based on the cost savings realized in a previous MDPH interventional study, Reducing Ethnic/Racial Asthma Disparities in Youth (READY) (11). Inclusion criteria for our study population, drawn from the MassHealth data warehouse, were similar to those for the children in a cohort (N = 22) of READY participants that had uncontrolled asthma (identified based on 2 or more ED visits during a 1-year period before the start of the asthma home visiting intervention). We used the aggregate and previously published findings from the READY study cohort to estimate the decrease in costs for asthma-related hospitalizations and ED visits associated with hypothetical CHW model expansion in our study population (11). Although the CHW model can take many forms, our analysis focused primarily on the model as it was applied in the READY study. The READY study was an intervention that evaluated the health and economic effects of the CHW model in Boston and Springfield, Massachusetts, among a population that was 93.3% insured by MassHealth. Ours was a simulation study of a subset of MassHealth-eligible children with uncontrolled asthma statewide. In the READY study, CHWs led in-home asthma management and environmental trigger-remediation education over 5 visits spanning 6 months, plus a follow-up telephone call at 12 months. CHWs provided asthma self-management education (eg, proper inhaler technique), environmental trigger remediation education (eg, green cleaning, integrated pest management practices), and low-cost trigger remediation supplies (eg, HEPA vacuum cleaners, mattress covers). Unlike the READY study, our study was not an intervention (ie, no asthma home visits were conducted). Instead, we estimated the impact of expanding the CHW model used in the READY study to a broader population of MassHealth-eligible children with uncontrolled asthma.

### Measures

We determined average per-person health care costs to MassHealth in 2019 for our study population by using medical claims data for ED visits and hospitalizations where asthma was the primary or secondary diagnosis. These costs were then used to calculate expected health care expenditures if the CHW model were expanded to our study population. To establish the potential cost savings of a hypothetical large-scale expansion of the CHW Model in the Medicaid population, we used the published aggregated results from the READY study to estimate the expected reduction in asthma-related health care costs associated with CHW model expansion among our study population. The percentage reduction in asthma-related ED visit and hospitalization costs observed in the relevant cohort of the READY study was approximately 70% for ED visits and 51% for hospitalizations. These calculations were based on the change in asthma-related ED visit and hospitalization costs reported for the children in the selected cohort of the READY study for 1 year before and 1 year after the intervention. Among participants in the relevant cohort of that study, asthma-related hospitalization costs were $2,543.76 pre-intervention and $1,243.14 post-intervention. Asthma-related ED costs were $1,512.87 pre-intervention and $454.39 post-intervention. We used these cost savings to calculate the expected cost savings for our hypothetical CHW model expansion.

### Statistical analysis

We conducted a simulation analysis of a hypothetical expansion of the CHW model in our study population. This analysis assumed that the same percentage reduction in asthma-related hospitalization and ED costs observed in the READY study would occur if the CHW model was applied to the statewide MassHealth-eligible pediatric population with uncontrolled asthma. We did not use the original patient-level data set from the READY study in our analysis; instead, we used the published aggregated results from the READY study to project the estimated cost savings associated with model expansion.

We used SAS to perform all calculations and analyses. Descriptive statistics for our study population were calculated along with estimated cost savings. In the READY study, Mann–Whitney U tests were performed to compare changes in annual medical expenses before and after the application of the CHW model intervention (11).

## Results

Of the 546,466 children with MassHealth eligibility for 1 day or more in 2019, 78,641 (14%) were eligible for 365 days in 2019 (Figure). Of these, 9,785 (12%) had an asthma diagnosis in 2019. Of these 9,785 children with asthma and 365 days of eligibility in 2019, 1,367 (14 %) had 2 or more asthma-related ED visits in that same year. Our study population of 1,367 was distributed relatively equally across age categories (1 – 4 y, 26.0%; 5 – 8 y, 23.4%; 9 – 12 y, 22.8%; 13 – 17 y, 27.8%). Most were male (58.3%). Race was identified as 26.3% Non-Hispanic White, 10.1% Hispanic, and 7.9% Non-Hispanic Black, but was unknown for more than half of the study population (51.3%). (Table 1).

**Figure:**
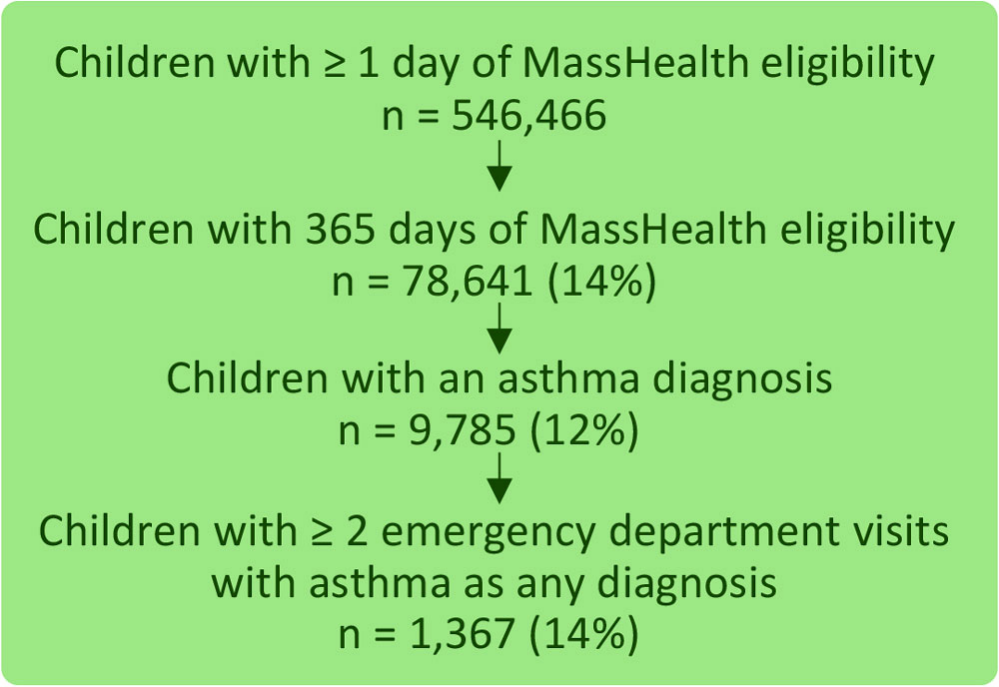
Sample selection results from 2019 MassHealth (Massachusetts Medicaid) medical and eligibility claims, accessed via their data warehouse.

**Table 1:** Demographic Characteristics, Participants With Asthma in the READY Study (N = 254)^a^ and in the Study of Massachusetts Medicaid (MassHealth)-Eligible Children With Uncontrolled Asthma (N = 1,367)^b^

Variable	READY study population^a^	MassHealth-eligible children with uncontrolled asthma^b^
Insurance type^c^		
Medicaid	237 (93.3)	1,367 (100.0)
Private	15 (5.9)^d^	0
Information missing	2 (0.8)^d^	0
Age, y		
1 – 4	Not reported	355 (26.0)
5 – 8		320 (23.4)
9 – 12		312 (22.8)
13 – 17		380 (27.8)
Age, y, mean	6.2	8.4
Sex		
Male	149 (58.7)	797 (58.3)
Female	105 (41.3)	570 (41.7)
Race or ethnicity^d^		
American Indian or Alaskan Native	Not reported	5 (0.4)
Asian or Pacific Islander	Not reported	15 (1.1)
Hispanic	125 (49.2)	138 (10.1)
Non-Hispanic Black	122 (48.0)	108 (7.9)
Non-Hispanic White	7 (2.8)	360 (26.3)
Multiracial	Not reported	40 (2.9)
Unknown	0	701 (51.3)

Abbreviation: READY, Reducing Ethnic/Racial Asthma Disparities in Youth.

a Massachusetts Department of Public Health study evaluating the health and economic effects of the community health worker model, a multicomponent, low-cost, asthma home visiting intervention for children with asthma, conducted in Boston and Springfield, Massachusetts, among a population that was 93.3% insured by MassHealth. Children who completed community health worker visit 1 were the only group of patients for whom demographic information was published (11). Values for some measures in the READY study were not published. Values are n (%) unless otherwise noted.

b Simulation study of hypothetical cost savings for a statewide subsample of children eligible for Massachusetts Medicaid (MassHealth) coverage in 2019, identified from MassHealth eligibility and medical claims extracted from the MassHealth data warehouse. The study population was defined as children with a full 365 days of MassHealth eligibility and at least 2 asthma-related emergency department visits in 2019 where asthma was the primary or secondary diagnosis.

c In the READY interventional study, insurance type was based on caregiver self-report. In the simulation study of statewide cost savings, all participants were MassHealth-eligible.

d Race and ethnicity data fields were largely incomplete in the MassHealth data warehouse.

The potential annual per-patient cost savings to MassHealth, post-expansion, for asthma-related ED visits in our study, calculated by taking the estimated annual per-patient cost of $490.69 and applying a 70.0% reduction, was $147.38, a total per-patient cost savings of $343.31 (Table 2). The estimated annual cost savings for asthma-related hospitalizations post-expansion, calculated by taking the estimated asthma-related hospitalization per-patient cost of $436.68 and applying a 51.1% reduction, was $213.41 per patient, a savings of $223.27. The combined (ED and hospitalization) asthma-related cost savings for pediatric MassHealth eligible patients with uncontrolled asthma were estimated to be $566.58 per patient or a total of $774,514.86 applied to the 1,367 children in our study.

**Table 2:** Estimated Pre-Intervention and Postintervention Costs, Study of MassHealth-Eligible Children With Uncontrolled Asthma (N = 1,367)^a^, Compared With Actual Costs of Children With Uncontrolled Asthma (N = 22) From the READY Study^b^

Study	Asthma-related ED visit costs per patient, 2019 US$		Asthma-related hospitalization costs per patient, 2019 US$	
	Pre-intervention	Postintervention	Pre-intervention	Postintervention^c^
READY study population^b^ (N = 22), mean	1,512.87	454.39	2,543.76	1,243.14
All MassHealth-eligible children^a^ (N = 1,367), mean	490.69	147.38	436.68	213.41

Abbreviations: ED: emergency department; READY, Reducing Ethnic/Racial Asthma Disparities in Youth.

a Study of hypothetical cost savings for a statewide subsample of children eligible for Massachusetts Medicaid (MassHealth) coverage in 2019 identified from MassHealth eligibility and medical claims extracted from the MassHealth data warehouse. The study population was defined as children with a full 365 days of MassHealth eligibility and at least 2 asthma-related emergency department visits in 2019 where asthma was the primary or secondary diagnosis (11). If a patient in the population of MassHealth-eligible children with asthma did not have an asthma-related hospitalization, their asthma-related hospitalization costs were considered 0. Costs of 0 were included when calculating the mean asthma-related hospitalization cost value.

b Massachusetts Department of Public Health study evaluating the health and economic effects of the CHW model, a multicomponent, low-cost, asthma home visiting intervention for children with asthma, conducted in Boston and Springfield, Massachusetts among a population that was 93.3% insured by MassHealth (Massachusetts Medicaid) (11). Pre-intervention costs for the READY study population were defined as the costs for a 1-year period before the first CHW visit in the intervention. Post-intervention costs were defined as the costs for a 1-year period starting after the last CHW visit of the intervention.

c Post-intervention costs for the overall MassHealth pediatric population were estimated based on the assumption that the proposed intervention (ie, the CHW-led asthma home visiting model) would lead to cost reductions proportional to those observed in the uncontrolled asthma cohort of the READY study. MassHealth medical claims for 2019 were obtained from the MassHealth data warehouse (an internal resource).

## Discussion

By using 2019 MassHealth claims data and combining it with results from the READY study, we estimated that over a 1-year period, $566.58 per patient or a total of $774,514.86 (in 2019 US dollars) in MassHealth expenditures could be saved across the 1,367 children in our study group through expansion of the CHW model. Although past analyses have estimated the cost savings associated with the CHW model in studies of small cohorts, ours is the first to our knowledge that estimates the potential savings across a broad population of Medicaid-eligible children with asthma. Our analysis adds to previous findings from the READY study that the CHW model would have important benefits for pediatric asthma outcomes (eg, a reduction in the average number of asthma symptom days, an increase in the percentage of children with well-controlled asthma, reduced exposure to environmental triggers) (11). Although the READY study included only families from Boston Medical Center and Baystate Medical Center in Springfield rather than a representative sample of the entire MassHealth population, our study population and the READY study population were largely comparable. Although 100% of our study group were MassHealth-eligible patients, only 93.3% of the READY study population were. Beyond this, we saw minor differences between the average age of the READY study population and ours (READY study, 6.2 years; our study, 8.4 years). As previously noted, race and ethnicity data available in MassHealth claims are largely incomplete, which restricted our ability to compare the racial breakdown of the READY study population and ours. Additionally, we could not adjust for differences in the READY study population and ours because we could not access individual patient data from the READY study.

Although the racial and ethnic composition of the 1,367 participants in our analysis is largely unknown, we did not use a stand-in variable, such as income or socioeconomic status. As acknowledged across leading public health frameworks and shown by a robust body of literature, race and racism affect health outcomes such as asthma, independent of the association between race and other socioeconomic factors such as income and cultural differences (8,22–24).

Efforts were made to validate these results, whenever possible. For example, based on available data on higher rates of asthma prevalence among residents of Massachusetts with low incomes (25), we expected that asthma prevalence in this MassHealth population would be slightly higher than that of the general population. This was the case when we compared our 12% asthma prevalence estimate from the MassHealth claims data with the 2019 Massachusetts statewide BRFSS asthma prevalence estimate of 9.5% (26). This finding helped establish the face validity of our MassHealth asthma prevalence estimate, which informed our cost projection estimates.

### Limitations

Our study had several limitations. Because of the resource-intensive nature of a large-scale expansion of the CHW model across our study population, our study only simulated the effects of this expansion (our proposed intervention was not conducted in our study population). First, as a hypothetical intervention, our results may differ in actual application. Second, estimates of cost savings may not extend to a different year, state, or patient population or to costs associated with a different insurance provider, potentially limiting the generalizability of our findings. Third, we applied data from the primarily Black and Hispanic READY study population to a population in which race was largely unknown. However, given the highly adaptable nature of the CHW model to the unique, lived experiences of the patients and communities served, we anticipate that applying the CHW model to a population with a different racial or ethnic composition would produce similar results. The program structure and implementation (eg, CHW staffing, training, level of CHW integration into primary care teams) also may vary across specific applications of the CHW model in ways that affect the benefit derived from the intervention. For example, interventions that are fully staffed by CHWs, receive adequate funding, and have more complete integration of CHWs into the primary care team may be associated with greater reductions in asthma-related health care use and greater cost savings.

An additional limitation of our study design is the assumption that all children covered by MassHealth with uncontrolled asthma would benefit equally from the CHW model. We recognize that some children may not benefit from this intervention for systemic, cultural, or patient-specific reasons (eg, earned distrust in the medical establishment, inflexible caretaker work schedules, other personal barriers that limit participation). Beyond this, how we define benefit — as a quantified measure of cost within the health care system — also has its limitations, because our definition fails to acknowledge the other less easily quantifiable emotional or social benefits that affect quality of life and that have been demonstrated with the CHW model (27).

Another important limitation is that our analysis did not factor in the costs associated with scaling up the CHW model. Instead, we focused solely on the reduction in asthma-related ED and hospitalization costs. Given that the costs of CHW model expansion can vary greatly over time and across communities, depending on a variety of factors (eg, staffing needs, unit costs of supplies, transportation costs), estimating the costs of expansion was outside the scope of our analysis. Our study only provides an estimate of the expected reduction in health care costs associated with the expansion and is not meant as a cost–benefit analysis. It may be useful for future researchers to build on this analysis to develop a more inclusive estimate of the cost savings associated with the expansion of the CHW model by factoring in costs associated with expansion.

Although our study had several limitations, we aimed to be as conservative as possible when estimating expected health care cost reductions, and we may have underestimated savings. First, we may have underestimated the size of the population that would be affected by this intervention. We defined children with uncontrolled asthma as those with 2 or more asthma-related ED visits per year in which asthma was the primary or secondary diagnosis, excluding children with asthma listed only as part of an additional diagnosis. We also made a full 12 months of MassHealth eligibility in 2019 an inclusion criterion, excluding those with less time of eligibility.

Beyond the conservative estimates of the patient population, we also conservatively estimated included costs. Restricting the health care costs considered in this analysis to only ED visits and hospitalizations where asthma was the primary or secondary diagnosis potentially excluded visits that were asthma-related, leading to an underestimation of costs that could be reduced by the CHW intervention. In addition, children with asthma may also have family members with asthma who could benefit from the intervention but whose cost savings would not have been captured in our analysis (13). We did not include pharmacy claims because they were incomplete in our data set. We also did not include the cost of clinician visits because we did not find them to be meaningfully affected by the CHW model in the READY study. Greater reductions in health care costs were observed in other similar studies by Gomez et al (15) and Campbell et al (12), which included other types of health care costs (eg, clinician visits, medication) associated with the CHW model, suggesting that our narrower focus on reductions in asthma-related ED visit and hospitalization costs may have led to more conservative estimates of cost savings associated with the CHW model. However, based on the inequities that we have identified in use of these health care services in Massachusetts, we feel that the exclusive use of asthma-related ED visits and hospitalizations as measures of health care use was reasonable. Despite our narrow focus, we believe that the identified reduction in asthma-related ED and hospitalization costs alone offers sufficient support for significant expansion of the CHW model.

Although we did not see a significant increase in clinician visit costs in the READY study, at a broader level health care use may shift toward an increase in primary care visits and their associated costs, which could offset some of the cost savings reported here. However, we anticipate that any such shifts would be minor and would not substantively affect the cost savings associated with this intervention from a clinical perspective, because the shift could indicate better asthma control.

### Conclusion

Evidence strongly suggests that large-scale expansion of the CHW model to children with uncontrolled asthma who are eligible for Medicaid would lead to cost savings. This expansion is also expected to improve asthma outcomes for the MassHealth population of children with uncontrolled asthma (11,13,14), which disproportionately contains Black and Hispanic children (28). Expansion of the CHW model is one part of a comprehensive approach to asthma care that has the potential to reduce racial disparities in asthma outcomes by increasing access to linguistically and culturally appropriate asthma care.
